# Transcriptome Reveals the Effects of Early Weaning on Lipid Metabolism and Liver Health of Yangtze Sturgeon (*Acipenser dabryanus*)

**DOI:** 10.3390/ijms231810866

**Published:** 2022-09-17

**Authors:** Xin Zhang, Youlian Liu, Shuhuang Chen, Bin Wang, Hongwei Wu, Ni Tang, Liulan Zhao, Song Yang, Qiao Liu, Bo Zhou, Defang Chen, Zhiqiong Li

**Affiliations:** 1 Department of Aquaculture, College of Animal Science and Technology, Sichuan Agricultural University, Chengdu 611130, China; 2 Fisheries Institute, Sichuan Academy of Agricultural Sciences, Yibin 644000, China

**Keywords:** lipid metabolism, liver health, weaning, Yangtze sturgeon (*Acipenser dabryanus*)

## Abstract

The Yangtze sturgeon (*Acipenser dabryanus*) has recently been declared extinct in the wild, and artificial breeding is the only means to protect its germplasm resources, but it has difficulty in weaning (from live prey to artificial food). In this study, we first performed a histological observation, enzyme-activity determination, and transcriptome sequencing on the livers of juvenile Yangtze sturgeons, and we then cloned five critical genes of lipid metabolism according to the transcriptome-sequencing results. We designed a weaning experiment to analyze their expression levels during weaning. The results showed that the density of hepatocytes and the transaminase activity of the juveniles failed to wean. The differentially expressed genes were enriched significantly in the pathways involving steroid synthesis, amino acid metabolism, and pancreatic secretion. It was found that the mRNA level of the fatty acid-synthesis gene decreased, and the mRNA level of the lipolysis gene increased significantly during weaning. The results of this research indicated that weaning could affect the liver health of Yangtze sturgeon, and it could affect the liver lipid metabolism by inhibiting fatty acid synthesis and promoting lipolysis. This study enhances our understanding of the impact of weaning on the lipid metabolism in fish.

## 1. Introduction

Weaning is commonly considered as the switch from one type of feed to another type, and it is a necessary stage for raising animals, including mammals and fish, to meet their nutritional requirements for growth. Fish, as a high-quality protein source of cultured animals, is becoming more and more popular, and with the promotion of intensive farming, aquaculture has become the fastest growing sector in the field of animal breeding [1]. In the process of aquaculture, weaning is a very critical moment for cultured fish due to the fact that the nutrition of live prey is not balanced enough to meet the needs of growth and survival. However, it is difficult for many fish to complete the weaning, which has become a bottleneck that is restricting the further development of aquaculture [2]. To solve this problem, scholars have carried out a lot of research. Previous studies have shown that weaning can affect the growth performance [3,4] and physiological functions of fish, including the digestion [2,5,6], innate immunity [7], and metabolism [8], but the mechanism is still unknown.

As one of the important links in aquaculture, there is a growing interest in the impact of weaning on fish physiology. The use of an inert diet instead of live prey changed the expressions of the digestive enzymes of spotted rose snapper (*Lutjanus guttatus*) [9] and glass eels (*Anguilla anguilla*) [10]. Moreover, the gut microbial community of the host is also affected by weaning [2]. Campoverde et al. [7] found that the innate immune gene-expression profiles (complement c3, Cyclooxygenase, Lysozyme, Mx protein, Myeloid differentiation primary response gene 88, NOD-like receptors) of juveniles also changed after weaning. In order to understand the mechanism of the effect of weaning on fish physiology, a few studies have used transcriptome technology and have found that weaning affects amino acid metabolism and lipid metabolism in fish, such as *Siniperca chuatsi* [8,11]. In addition, previous studies have shown that feed substitution has an effect on fish lipid metabolism, but these studies mainly focus on the impact of feed nutrition [12,13,14,15], and there is a lack of research on the impact of weaning on fish lipid metabolism.

Sturgeon is one of the earliest vertebrates in existence, which are known as living fossils, many of which are endangered and listed as first-class protected wild animals, such as the Yangtze sturgeon (*Acipenser dabryanus*) and Chinese sturgeon (*Acipenser sinensis*) [16]. So far, there have been research reports on the weaning of some sturgeons, such as Siberian sturgeon (*Acipenser baerii*) [17], Persian sturgeon (*Acipenser persicus*) [18], sterlet (*Acipenser ruthenus*) [19,20], white sturgeon (*Acipenser transmontanus*) [21], Atlantic sturgeon (*Acipenser oxyrinchus*) [22], and Yangtze sturgeon [23]. Nevertheless, these studies were mainly concerned with growth, survival, and intestinal microbes, and there is no report on the effect of weaning on the physiological metabolism of sturgeon. Yangtze sturgeon, the flagship species for the protection of rare and endemic fish in the upper reaches of the Yangtze River, is a migratory fish that is distributed only in the middle and upper reaches and tributaries of the Yangtze River in China [24], and it has been listed in Appendix II of the Convention on International Trade in Endangered Species of Wild Fauna and Flora (CITES) due to the decline of wild resources. Since 2000, no naturally bred juvenile fish have been found [25], and so artificial cultivation is a necessary means to protect and restore its germplasm resources. During artificial cultivation, weaning is an important challenge to be overcome for Yangtze sturgeon. However, research on the weaning of Yangtze sturgeon is scarce, and the effect of weaning on the physiological function of Yangtze sturgeon and the mechanism are still unclear.

In order to explore the effect of weaning on Yangtze sturgeon, juvenile Yangtze sturgeons that failed and succeeded in weaning were selected for anatomical observation, and the histology and enzyme activities of the livers were detected due to the atrophy of the livers of the weaning failed larvae. Next, RNA-Seq was used to investigate the effect of weaning on the liver. Subsequently, based on the results of the RNA-Seq, the cDNA of several critical genes of lipid metabolism were cloned, including acetyl-CoA carboxylase-1(*ACC1*), fatty acid synthase (*FAS*), carnitine palmitoyl transferase 1(*CPT1*), sterol-regulator element-binding protein 1(*SREBP1*), and peroxisome proliferators activate receptors γ (*PPARγ*). The expressions of these genes and adipose triglyceride lipase (*ATGL*) during weaning were further detected to understand the effect of weaning on lipid metabolism. These genes are closely related to lipid metabolism in fish: *ACC1* and *FAS* function in fatty acid biosynthesis, *ATGL* and *CPT1* play key roles in the process of lipolysis, and *SREBP1* and *PPARγ* play important roles in lipid metabolism as transcription factors [26]. This study is expected to provide a theoretical basis for formulating reasonable weaning strategies for Yangtze sturgeon to contribute to the conservation and recovery of the germplasm resources of Yangtze sturgeon, and to increase our understanding of the physiological and metabolic mechanisms of weaning in the teleost.

## 2. Results

### 2.1. Histological Observation and Transaminase Activity of Liver

**Figure 1:**
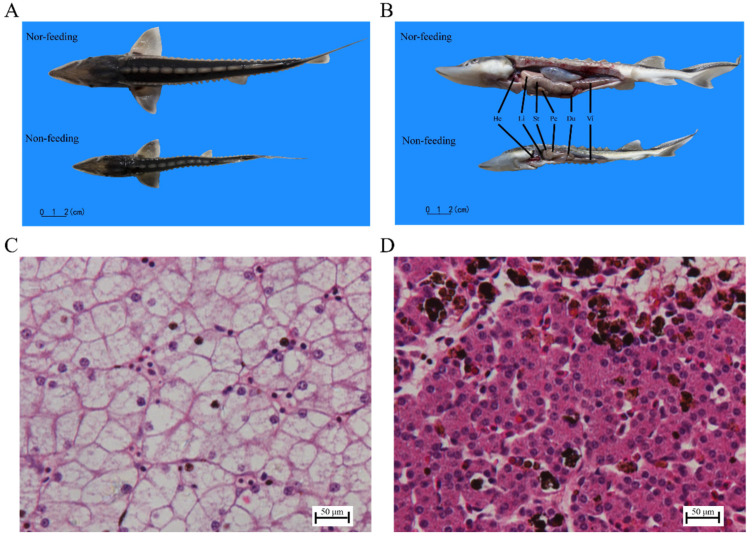
Weaning affected the liver health of Yangtze sturgeons. (**A**,**B**) Comparison of morphology and anatomy of Yangtze sturgeons. He: heart; Li: liver; St: stomach; Pc: pyloric caeca; Du: duodenum; Vi: valvula intestine. Images of HE-stained liver sections in (**C**) successful and (**D**) failed weaning Yangtze sturgeons (40×). Nor-feeding: successful weaning; non-feeding: failed weaning.

Compared with the nor-feeding fish, the non-feeding fish were thin, the livers were atrophic, and there was no content in the intestines (Figure 1A,B). The liver-tissue sections of the non-feeding and nor-feeding fish were observed under optical microscopes. The results showed that the hepatocytes of the nor-feeding fish had clear boundaries, the nucleus was located at the cell edge, and the cytoplasm was lightly stained, but there was mild congestion in the central vein and hepatic sinuses (Figure 1C). In contrast, the hepatocytes of the non-feeding fish were closely arranged, the boundaries were not clear, the cytoplasm was deeply stained, hemosiderin was deposited around the sinuses, Kupffer cells, which are a marker of liver health, increased, and the hepatic sinusoids dilated (Figure 1D).

**Figure 2:**
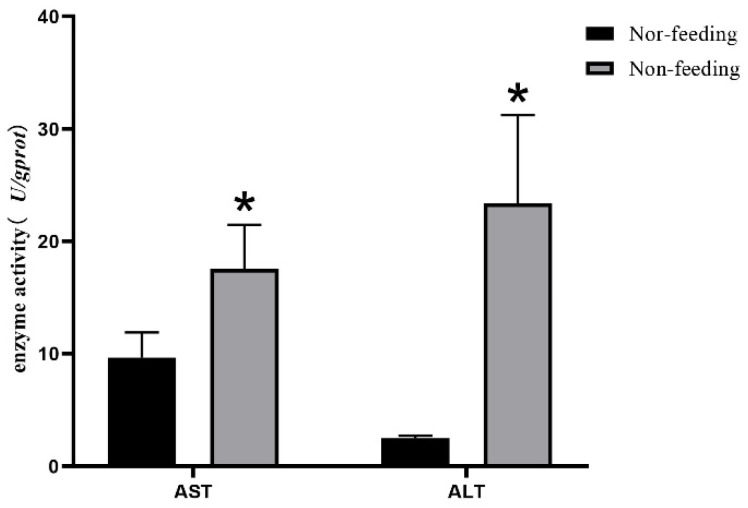
Activity of aspartate aminotransferase and alanine transaminase in liver. Nor-feeding: successful weaning; non-feeding: failed weaning. * *p* < 0.05.

Compared with the Success-Fish, the activities of aspartate aminotransferase (AST) and alanine aminotransferase (ALT) in the liver of the Failure-Fish increased significantly (*p* < 0.05) (Figure 2).

### 2.2. Transcriptome-Data Analysis

The F_L and S_L groups obtained 54,101,748 and 64,480,944 raw reads, respectively. After treatment, 53,678,894 and 63,958,528 clean reads were obtained from the F_L and S_L groups, respectively, with Q20 (nucleotides with quality values > 20) > 98%, Q30 > 94%, and an error rate <0.025%. The quality control is qualified and can be used for subsequent analysis (Table 1). These clean reads were assembled into 78,286 transcripts with an N50 length of 1740 bp, and a final set of 52,559 unigenes with an N50 length of 1613 bp was generated. The scores for the quality assessment of the transcript assembly using TransRate and BUSCO were 0.43717 and 95.3%, respectively (Table 2).

**Figure 3:**
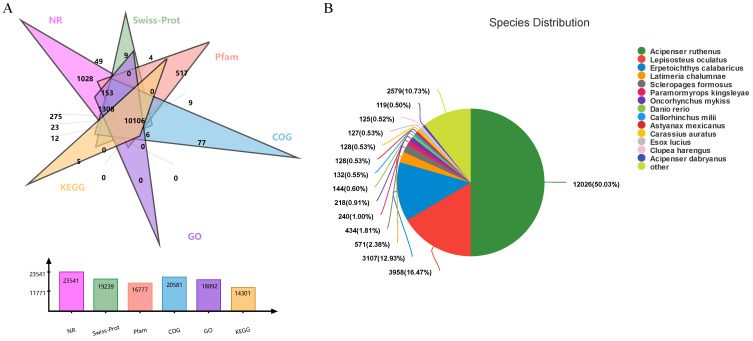
Distribution of the annotated genes: (**A**) unigene-function-annotated Venn diagram; (**B**) NR-annotated pie chart of species distribution.

**Figure 4:**
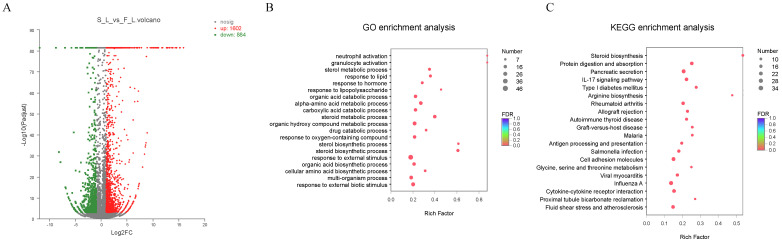
Differentially-expressed-gene screening and enrichment analysis: (**A**) volcano map of differentially expressed genes in livers; (**B**) GO enrichment; (**C**) KEGG-pathway enrichment of DEGs in livers.

In order to obtain annotation information, the 52,559 unigenes were annotated in six databases, including NR, SwissProt, Pfam, COG, GO, and KEGG, after assembly optimization (Figure 3A). The results showed that 23,541 (46.38%), 19,239 (37.91%), 16,777 (33.05%), 20,581 (40.55%), 18,892 (37.22%), and 14,301 (28.18%) unigenes were annotated in the above databases, respectively (Figure 4A). Moreover, 12,026 clusters (50.03%) were similar to *Acipenser ruthenus*, 3958 clusters (16.47%) were similar to *Lepisosteus oculatu*, and 3107 clusters (12.93%) were similar to *Erpetoichthys calabaricus* (Figure 3B).

The screening results of the differentially expressed genes showed that 2486 differentially expressed genes were screened in the livers, of which 1602 were upregulated and 884 were downregulated (Figure 4A). According to the expressions of the DEGs, and for the sake of further studying the effect of weaning on the transcription level of juvenile Yangtze sturgeon, the upregulated and downregulated genes in the livers were enriched by GO. Figure 4B shows the enriched biological processes that were involved: the steroid metabolic process, steroid biosynthetic process, cellular amino acid biosynthetic process, and organic hydroxy compound metabolic process (*p* < 0.05). The results of the KEGG enrichment of the DEGs in the livers showed they were mainly enriched in steroid biosynthesis, arginine biosynthesis, glycine, serine, and threonine metabolism, as well as pancreatic secretion (Figure 4C) (*p* < 0.05).

**Figure 5:**
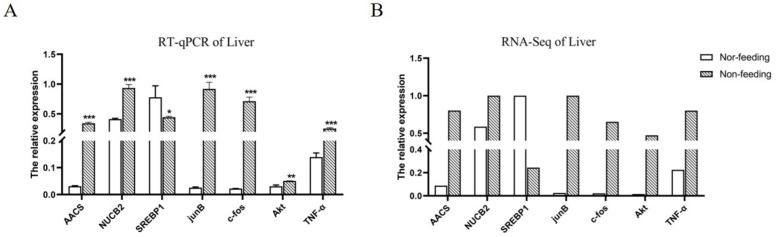
Part of DEGs were verified by RT-qPCR. Gene-expression levels were normalized to that of *β-actin* and *EF1-α*. Data are presented as the group means ± SD (*n* = 5). Statistical comparison of the mRNA levels detected in different groups was carried out by *t*-test of variance (* *p* < 0.05, ** *p* < 0.01, *** *p* < 0.001). (**A**) RT-qPCR of liver; (**B**) RNA-Seq of liver.

In order to verify the expressions of the RNA-Seq analysis, a total of seven genes in the livers were verified by qPCR at the mRNA level. The results showed that the expression trends of these genes were the same under the detection of qPCR and RNA-Seq. The expressions of *AACS*, *NUCB2*, *JunB*, *c-fos*, *Akt*, and *TNF-α* were significantly upregulated, and the expression of *SREBP1* was significantly downregulated (Figure 5).

### 2.3. Gene Cloning and Sequence Analysis

**Figure 6:**
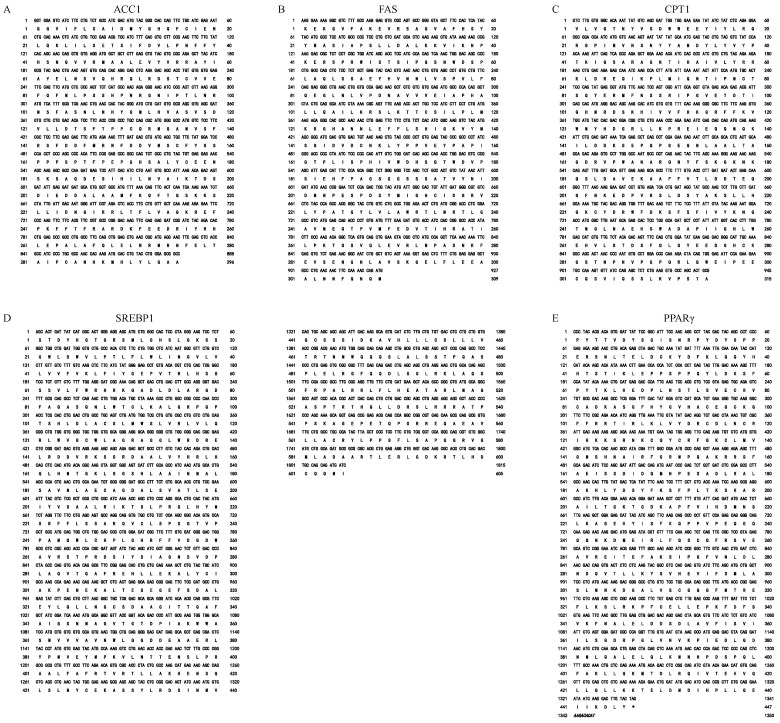
cDNA sequences and deduced amino acid sequences of (**A**) *ACC1*, (**B**) *FAS*, (**C**) *CPT1*, (**D**) *SREBP*, and (**E**) *PPARγ* in the Yangtze sturgeons. The asterisk (*) indicates the stop codon.

To investigate the effect of weaning on the lipid metabolism of Yangtze sturgeon, the key factors of lipid metabolism (*ACC1*, *FAS*, *CPT1*, *SREBP1*, and *PPARγ*) were cloned from the livers of Yangtze sturgeons. The amplified parts of the coding sequences (CDSs) of *ACC1*, *FAS*, *CPT1*, *SREBP1*, and *PPARγ* were 888, 927, 945, 1815, and 1350 bp in length (Figure 6A–E), respectively, encoding for a putative protein of 296, 309, 315, 605, and 447 amino acids, respectively. The nucleotide sequences of these genes of Yangtze sturgeon were submitted to GenBank with the GenBank numbers: MN685787, MN685791, MN685789, MN685809, and MN685800.

Based on the deduced ACC1, FAS, CPT1, SREBP1, and PPAR*γ*, a multiple sequence analysis of the amino acids showed that these amino acid sequences of different species were highly conserved (Figure S1). The consistency of the ACC1 amino acid sequence between *Acipenser dabryanus* and other species was more than 75% (Figure S1), among which the consistency with the ACC1 amino acid sequence of *Lepisosteus oculatus* was the highest (91.9%), followed by *Gallus gallus* (86.8%), and the lowest was with *Danio rerio* (75.9%). The consistency of the FAS amino acid sequence between *Acipenser dabryanus* and *Acipenser ruthenus* was the highest (96.1%), followed by *Cyprinus carpio* (83.5%), and the lowest was with *Sus scrofa* (61.3%) (Figure S2). The consistency of the CPT1 amino acid sequence between *Acipenser dabryanus* and *Lepisosteus oculatus* was the highest (83.9%), followed by *Gallus gallus* (78.7%), and the lowest was with *Mus musculus* (73.9%) (Figure S3). SREBP1 was similar to FAS (Figure S4). The consistency of the PPAR*γ* amino acid sequence between *Acipenser dabryanus* and *Acipenser ruthenus* was the highest (98.4%), followed by *Homo sapiens* (81.2%), and the lowest was with *Salmo solar* (60.0%) (Figure S5).

**Figure 7:**
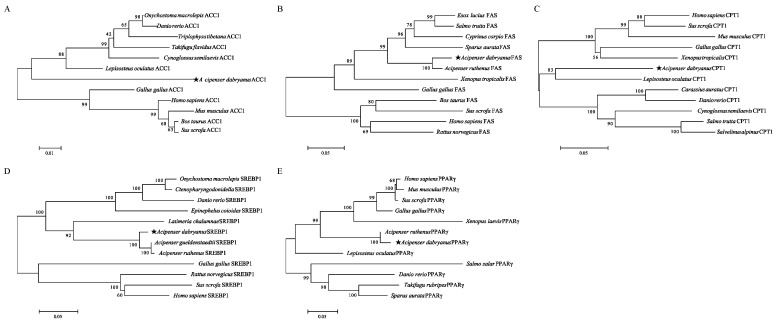
Phylogenetic trees of (**A**) *ACC1*, (**B**) *FAS*, (**C**) *CPT1*, (**D**) *SREBP*, and (**E**) *PPARγ* ammonia acid sequences in Yangtze sturgeons. Numbers at nodes indicate the boot-strap values, as percentages, obtained for 1000 replicates. Yangtze sturgeon is marked with a pentagram (★).

The evolutionary relationships of these genes are shown in Figure 7. The *ACC1* was mainly classified into two clades, with mammals as an outgroup. The genetic relationship between *Acipenser dabryanus FAS* and *Acipenser ruthenus FAS* was the closest, and the farthest from mammals (human, rat, and cow, etc.), which was consistent with the results of the animal classification. The genetic relationship between *Acipenser dabryanus CPT1* and *Lepisosteus oculatus CPT1* was the closest. *SREBP1* was similar to ACC1. The genetic relationship between *Acipenser dabryanus PPARγ* and *Acipenser ruthenus PPARγ* was the closest (Figure 7A–E).

### 2.4. Effects of Weaning on the Expressions of Genes Related to Liver Lipid Metabolism

**Figure 8:**
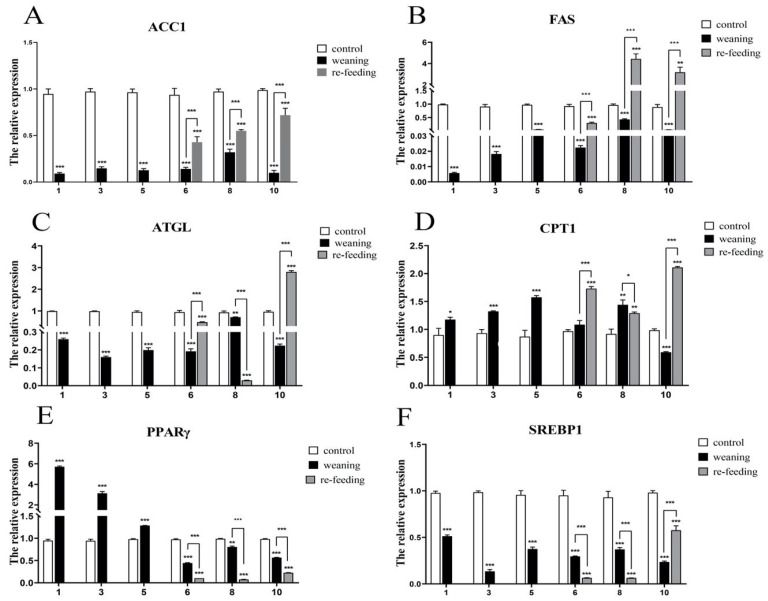
Weaning and refeeding earthworms induced changes in mRNA levels of (**A**) *ACC1*, (**B**) *FAS*, (**C**) *ATGL*, (**D**) *CPT1*, (**E**) *PPARγ* and (**F**) *SREBP1*. Gene-expression levels were normalized to that of *β-actin* and *EF1-α*. Data are presented as the group means ± SD (*n* = 5). Statistical comparison of the mRNA levels detected at different groups was carried out by *t*-test of variance (* *p* < 0.05, ** *p* < 0.01, *** *p* < 0.001).

The effect of weaning on the key enzymes and regulatory factors of lipid metabolism in the liver is shown in Figure 8 Compared with the control group, the expressions of the key enzymes in fatty acid synthesis (*ACC1* (Figure 8A) and *FAS* (Figure 8B)) in the weaning group declined in 10 days (*p* < 0.001). After refeeding, the expressions of *ACC1* and *FAS* were significantly higher than the weaning group (*p* < 0.001), and the expression of *FAS* was significantly higher than the control group on the 8th (*p* < 0.001) and 10th (*p* < 0.01) days. The expression pattern of *ATGL* (cloned but unpublished) was similar to *FAS*; however, the expression of *ATGL* was significantly lower than that in the weaning group on the 8th day (*p* < 0.001) (Figure 8C). The expression changes of *CPT1*, the key enzyme of lipolysis, are shown in Figure 8D. Compared with the control group, the expression of *CPT1* in the weaning group increased significantly (days 1–8) (*p* < 0.05) and decreased on the 10th day (*p* < 0.001). After refeeding, the expression of *CPT1* was significantly higher than that in the control group and weaning group (*p* < 0.01); however, the expression of *CPT1* was significantly lower than that in the weaning group on the 8th day (*p* < 0.05). Compared with the control group, the lipid-metabolism regulator *PPARγ* increased significantly in the early stage (days 1–5) (*p* < 0.001), but it decreased significantly in the later stage (days 6–10) (*p* < 0.01), showing a time-dependent trend. After refeeding, its expression was significantly lower than that in the weaning group (*p* < 0.001) (Figure 8E). The expression of *SREBP1* (Figure 8F), another metabolic regulator, decreased significantly after weaning (*p* < 0.001), and continued to decrease after refeeding (*p* < 0.001). Moreover, on the 10th day, it was significantly higher than that in the weaning group, and significantly lower than that in the control group (*p* < 0.001).

## 3. Discussion

In this study, the Yangtze sturgeons that failed to wean were significantly thinner, and the body lengths and weights were significantly lower than those of the successful weaning juveniles [27]. After dissecting the Yangtze sturgeons, it was found that the failure in weaning caused liver and heart atrophy in the Yangtze sturgeons. As an important organ of metabolism, the histomorphology of the liver can reflect the physiological disorder caused by the imbalance or deficiency of the feed nutrition. ŽáK et al. [5] found that male turquoise killifish possessed a lipid type of hepatocellular vacuolation after using commercial feed instead of live prey. This study was the first to compare the livers of failed and successful weaning juveniles, and it found that the hepatocyte density of juvenile fish with failed weaning increased, and the boundary of the cell membrane was blurred. Kupffer cells also increased in the liver of juveniles with failed weaning. Kupffer cells are macrophages that reside in the liver and are involved in many important physiological and pathological processes, including inflammation, fatty liver, and tumor [28,29]. The results of the liver-tissue sections further confirmed that the failure to wean could damage liver health. In addition, the activities of ALT and AST in the liver of juvenile fish with failed weaning in this study also increased significantly. ALT is not only a metabolic enzyme related to energy metabolism, but also a marker of liver damage [30]. AST is closely related to protein metabolism and is an importantly sensitive index in response to tissue and cell damage [31]. These results were consistent with the tissue-section results, which further confirmed that weaning could affect the liver health of Yangtze sturgeon.

In order to further understand the mechanism of weaning affecting liver health, the RNA-Seq was taken to determine the transcription profile in the livers of the successful and failed weaning Yangtze sturgeons. Zhao et al. [2] obtained 42,631 unigenes and 867 DEGs from the livers of largemouth bass larvae at different stages of weaning using high-throughput sequencing. The genes with increased expression were related to amino acid metabolism in the preweaning stage, but they were related to fatty acid metabolism in the postweaning stage. He et al. [8] found three pathways related to food-habit domestication through transcriptome and metabolome analysis, including retinol metabolism, glycerin metabolism, and unsaturated fatty acid biosynthesis. These studies showed that weaning can affect different physiological metabolism processes of fish. The present results indicated that there were 78,286 transcripts and 52,559 unigenes, of which 2486 were DEGs. It was found that the DEGs in the livers were significantly enriched in the pathways involved in steroid biosynthesis, amino acid metabolism, and pancreatic secretion. These results indicate that weaning affects the liver metabolism, and they lay a foundation for a follow-up study of the effect of weaning on liver lipid metabolism.

As the RNA-Seq results suggested that lipid metabolism would be affected by weaning, the partial cDNA of *ACC1*, *FAS*, *CPT1*, *SREBP1*, and *PPARγ* were cloned from Yangtze sturgeons to further explore the effect of weaning on lipid metabolism. The cloning of these genes has been reported in some fish, including grass carp (*Ctenopharyngodon idella*), yellow catfish (*Pelteobagrus fulvidraco*) [32], and orange-spotted grouper (*Epinephelus coioides*) [33]. In the study, the amino acid-sequence analysis showed that these genes were highly consistent among different species, and the phylogenetic-analysis results based on the amino acid sequence was consistent with the traditional classification and evolutionary status. This study lays a foundation for the follow-up study of lipid metabolism in fish.

Based on the results of the transcriptome sequencing and gene cloning, we explored the effect of weaning on lipid metabolism. An important component of lipid metabolism, including in fish, is the biosynthesis of fatty acids [34]. ACC1 and FAS play a key role in the process of fatty acid synthesis. ACC1 relies on biotin to catalyze the irreversible carboxylation of acetyl-CoA to malonyl-CoA, which is the rate-limiting step in the pathway of fatty acid biosynthesis [35]. FAS can convert acetyl-CoA and malonyl-CoA into palmitate, and then esterify it into triglyceride (TAG) stored in adipose tissue [36]. In this study, the mRNA levels of *ACC1* and *FAS* decreased in the weaning group, but the mRNA levels of *ACC1* and *FAS* increased after refeeding with *T. Limnodrilus*, indicating that the direct weaning of the Yangtze sturgeons inhibited their fatty acid synthesis. After refeeding with water earthworm, the synthesis of fatty acids increased, promoting fat deposition, which is an important reason for the weight gain of juvenile fish after refeeding. Peng et al. found that the expression of *FAS* of juvenile turbot was upregulated and promoted liver fat deposition after using soybean oil instead of fish oil [37]. For Atlantic bluefin tuna, compared with *Artemia*, the expression of *FAS* in the larvae was higher when they were fed with copepods with a high DHA and DHA/EPA ratio [12]. The reason for these differences may be that the purpose of these studies was to find fish-oil substitutes. This study explored the effect of weaning on lipid metabolism for the first time, which provides ideas for formulating reasonable weaning schemes in the future. This study shows that direct weaning can inhibit fatty acid synthesis by reducing the expressions of *ACC1* and *FAS*, and refeeding live prey can reduce this effect.

Moreover, lipolysis is another important process of lipid metabolism, which is crucial for maintaining the energy balance of fish. ATGL is an important part of lipolysis and the mobilization of the lipid stores in mammals. It can specifically hydrolyze the first ester bond of TG to form diglyceride (DG) and free fatty acid (FFA) [38], as it is the rate-limiting enzyme in the hydrolysis of TG. CPT1 is mainly distributed in the outer membranes of microsomes and mitochondria and is a fatty acid β-oxidation-limiting enzyme in the oxidation process [39]. These two enzymes play important roles in regulating lipid catabolism and energy metabolism. In the present study, the mRNA level of *ATGL* decreased during weaning, but recovered after refeeding with *T. Limnodrilus*, and it was higher than those of the other two groups at the 10th day. In large yellow croaker, after feeding with a high-fat diet, the expression of *CPT1* in the liver decreased significantly, whereas the expression of *ATGL* did not change significantly [15]. Li et al. [14] fed juvenile pond loach with different dietary soybean-oil levels, and they found that the expressions of the fatty acid-synthesis genes, such as *FAS*, increased, and fat-decomposition genes, such as *ATGL* and *CPT1*, decreased. In this study, the expression of *CPT1* mRNA increased first, and then decreased in the weaning group. After refeeding with *T. Limnodrilus*, the expression increased significantly. These results may be because n-3 LC PUFA in fish oil stimulates the expression of lipolytic factor CPT1 and enhances fatty acid β-oxidation [34]. These results showed that Yangtze sturgeons need to mobilize a large amount of fatty acid oxidation for their energy supply during weaning, due to the reduction in the energy intake, to increase the process of lipolysis. The results of the present study showed that direct weaning promoted lipolysis, and this effect was further enhanced when refeeding was performed.

## 4. Materials and Methods

### 4.1. Animals

The experimental fish were the F2-generation offspring of a captive-bred population of Yangtze sturgeons. Artificial reproduction of the fish was implemented at the facilities of the Fisheries Research Institute of Sichuan Academy of Agricultural Sciences, Sichuan, China. The larvae were fed chopped *T. Limnodrilus* beginning 4 days posthatching (dph), with natural light and filtered river water (20.5 ± 1.0 °C; changed twice a day, and one-third of the total volume of water was changed each time). All experimental procedures were approved by the Animal Care and Use Committee of SAAS and Sichuan Agricultural University (approval numbers: SAAS20180628 and DKY-S20180629).

### 4.2. Experimental Design and Sample Collection

During the breeding process of sturgeon seeds, there is a weaning stage (the live prey is changed to artificial feed). After being weaned for a period, some larvae fail to wean. In order to investigate the effect of weaning on the digestive system of Yangtze sturgeon, 10 juveniles failed to wean (characterized by a thin body, poor vitality, and less than 70% of the intestinal-filling degree after feeding for 30 min), and 10 juveniles that succeeded to wean were randomly selected. After low-temperature anesthesia, these fish were dissected rapidly for anatomical observation. The liver tissues of 5 fish in each group were frozen in liquid nitrogen and stored at −80 °C for transcriptome analysis and transaminase-activity detection. The liver tissues of the other 5 fish were fixed in Bonn’s solution for histological analysis.

Based on the results of the RNA-Seq, this study performed the gene cloning. For the cloning, 5 juvenile Yangtze sturgeons (224.56 ± 31.75 g) were randomly selected and hypothermically anesthetized. The liver tissues were quickly taken out, frozen in liquid nitrogen, and stored at −80 °C.

To investigate the effect of weaning on the lipid metabolism of Yangtze sturgeon, 360 juvenile Yangtze sturgeons were randomly divided into three groups: a control group, weaning group, and refeeding group. Each group was split into three tanks, with 40 fish per tank. At 60 dph, the control group continued to feed on *T. Limnodrilus*, while the weaning group was fed with commercial feed (the ingredients and chemical composition of the feed are shown in Table 3). The refeeding group was fed with commercial food and refed with *T. Limnodrilus* on the 5th day. Each group was fed at 8:00 and 20:00 every day to ensure fish satiety. At 14:00 on the 1st, 3rd, 5th, 6th, 8th, and 10th days after weaning, 6 sturgeons were randomly selected from each group to collect the livers for the subsequent experiments.

### 4.3. Histological Analysis of Liver

The liver was repaired to an appropriate size, fixed in neutral formalin, washed with water, dehydrated with gradient alcohol, made transparent, soaked in wax, and embedded in paraffin. The embedded tissue was cut into 3–5 μm slices. Then, hematoxylin–eosin (HE) staining was performed: the sections were fixed on slides, then dewaxed and rehydrated, stained with hematoxylin for 3 min, differentiated with 1% hydrochloric acid alcohol, rinsed with tap water, stained with eosin for 1 min, washed with tap water, dehydrated with ethanol gradient, clarified with xylene, and sealed with neutral gum. The tissue sections were observed and photographed with a Nikon Eclipse Ti-s microscope (Nikon, Tokyo, Japan).

### 4.4. Transaminase Activity in Liver

The livers were made into a tissue homogenate at a ratio of 1:9 with 0.9% saline solution, and the supernatant was obtained after centrifugation at 2500 rpm for 10 min. The total protein concentration was determined by the Coomassie Brilliant Blue method. The activities of ALT and AST were measured according to the instructions (Nanjing Jiancheng, Nanjing, China).

### 4.5. RNA Extraction and Transcriptome Sequencing

Total RNA was extracted from the livers of juvenile Yangtze sturgeons using the Spin Column Animal Total RNA Purification Kit (Sangon Biotech, Shanghai, China). The concentration of total RNA was detected by a bioanalyzer (Agilent 2100, Agilent Technologies, Santa Clara, CA, USA) and 1% agarose gel electrophoresis. RNA with RIN ≥ 7.0, OD 260/280 ≥1.8 and OD 260/230 ≥1.5 was approved for the next step.

The construction of the cDNA library and Illumina sequencing were completed by Majorbio (Majorbio, Shanghai, China). To ensure the reliability of the analysis, SeqPrep (https://github.com/jstjohn/SeqPrep (accessed on 8 July 2011)) and Sickle (https://github.com/najoshi/ sickle (accessed on 4 April 2012)) were used to filter the raw reads to remove the low-quality sequences, sequenced splice sequences, and repetitive redundant sequences (unknown nucleotides greater than 5%). After obtaining clean reads, Trinity (https://github.com/trinityrnaseq/trinityrnaseq (accessed on 25 May 2019).) was used for the de novo assembly to obtain the nonredundant unigenes.

The unigenes were compared with six databases to obtain the annotation information. The six databases are the following: NR (https://www.ncbi.nlm.nih.gov/refseq/about/nonredundantproteins/ (accessed on 24 August 2020)); Swiss-Prot (http://web.expasy.org/docs/swiss-prot_guideline. html (accessed on 8 December 2019)); Pfam (http://pfam.xfam.org/ (accessed on 27 September 2018)); COG (http://www.ncbi.nlm.nih.gov/COG/ (accessed on 25 November 2020).); GO (http://www.geneontology.org (accessed on 29 May 2019)); KEGG (http://www.genome.jp/kegg/ (accessed on 1 November 2018)).

RSEM (http://deweylab.github.io/RSEM/ (accessed on 27 June 2018)) was used for the transcriptome quantification. After the read counts between the different samples were standardized based on the TMP method, DEGseq software was used to screen the differentially expressed genes (DEGs) according to *p*-adjust < 0.001 and | log2FC | ≥ 2. The software Goatools (v1.0.15, Fuzhou, China) was used to perform the GO-enrichment analysis, and R language was used to write the scripts to perform the KEGG-pathway-enrichment analysis on the DEGs.

### 4.6. Gene Cloning

The total RNA of the livers was extracted using the Spin Column Animal Total RNA Purification Kit (Sangon Biotech, Shanghai, China). The cDNA was synthesized by the PrimeScript™ RT reagent kit with the gDNA Eraser (Takara, Dalian, China). According to the sequences of the teleost published on GenBank and the transcriptome sequencing, primers of *ACC1*, *FAS*, *CPT1*, *SREBP1*, and *PPARγ* were designed by Primer Premier 5.0 software (Table S1) and synthesized by Sangon Biotech (Shanghai, China). The PCR program was the same as a previous study [40]. The BLAST was used to analyze the similarity of the sequences. The deduced amino acid sequence was analyzed with Clustalx W (http://www.clustal.org/ (accessed on 13 January 2011)). A phylogenetic tree was constructed based on the amino-sequence alignment by the neighbor-joining (NJ) algorithm embedded in the MEGA 11 program (Philadelphia, PA, USA). The reliability of the branching was tested by bootstrap consensus (1000 replicates).

### 4.7. Quantitative Real-Time PCR (qRT-PCR)

In order to study the effect of weaning on the liver lipid metabolism of Yangtze sturgeon, the expressions of the key factors of lipid metabolism (*ACC1*, *FAS*, *ATGL*, *CPT1*, *SREBP1*, and *PPARγ*) were analyzed. The total RNA extraction and cDNA synthesis were the same as above. Primers (Table S1) with standard curve amplification efficiency (97~99%) and R^2^ (0.967~0.998) that meet the requirements can be used for subsequent tests. The results were normalized using *β-actin* and *EF1-α* as the internal control [41]. Real-time PCR assays were carried out on a quantitative thermal cycler (CFX Connect Real-Time System, BIO-RAD, Hercules, CA, USA). Gene expression was calculated using the comparative-threshold-cycle method (2^−△△CT^).

### 4.8. Statistical Analysis

SPASS 22.0 statistical software (SPSS Inc., Chicago, IL, USA) was used for statistical analysis. The data were presented as mean ± SD. An independent between-variable *t*-test was performed to determine the significant differences between the two groups. *p* < 0.05 was recorded as significant, and *p* < 0.01 was recorded as extremely significant.

## 5. Conclusions

In conclusion, this study found that the failure to wean could cause growth retardation and liver damage in juvenile Yangtze sturgeon. Moreover, through the RNA-Seq, it was found that weaning could affect steroid biosynthesis, amino acid metabolism, and pancreatic secretion in the liver. Moreover, the critical genes of lipid metabolism, including *ACC1*, *FAS*, *CPT1*, *SREBP1*, and *PPARγ*, were cloned in Yangtze sturgeon for the first time. Furthermore, it was found that direct weaning could inhibit fatty acid synthesis by decreasing *FAS* and *ACC1* and the nuclear transcription factors *SREBP1* and *PPARγ*, and could enhance lipolysis by stimulating the *CPT1* expression in the Yangtze sturgeon, while the expression of *ATGL* decreased. The results of the present study indicate that weaning can affect liver health and inhibit the growth of Yangtze sturgeon by reducing fatty acid synthesis and promoting lipolysis. This study provides a theoretical basis for a further understanding of the mechanism of weaning on fish physiological metabolism, and especially lipid metabolism. These genes can be utilized as target genes to develop appropriate weaning regimens and feed formulations.

## Figures and Tables

**Table 1:** Formulation and nutritional composition of commercial feed.

Sample	Raw Reads	Raw Bases	Clean Reads	Clean Bases	Error Rate (%)	Q20	Sample	Raw Reads
F_L	54,101,748	8,169,363,948	53,678,894	8,008,681,153	0.0244	98.33	94.72	48.04
S_L	64,480,944	9,736,622,544	63,958,528	9,527,555,685	0.0242	98.38	94.86	47.77

**Table 2:** Statistical table of assembly-result evaluation.

Type	Unigene	Transcript
Total number	52,559	78,286
Total base	46,907,651	77,903,931
Largest length (bp)	15,546	15,546
Smallest length (bp)	201	201
Average length (bp)	892.48	995.12
N50 length (bp)	1613	1740
E90N50 length (bp)	2675	2399
Fragment-mapped percent (%)	67.268	79.695
GC percent (%)	44.37	44.4
TransRate score	0.37572	0.43717
BUSCO score	C:95.3% [S:93.3%; D:2.0%]	C:95.3% [S:93.3%; D:2.0%]

**Table 3:** Statistical table of assembly-result evaluation.

Ingredients	Nutrient Composition (%)
Casein	49
Gelatin	5
α-starch	25
Fish oil	8
Choline chloride	0.25
Vitamin and mineral premix	1
CMC-Na	4
Microcrystalline cellulose	2.75
Potassium sorbate	0.1
Moisture	6.35
Crud protein	42.55
Crud lipid	10.53

## Data Availability

The original contributions presented in the study are included in the article. Further inquiries can be directed to the corresponding authors.

## Supplementary Materials

The supporting information can be downloaded at https://www.mdpi.com/article/10.3390/ijms231810866/s1.
