# Supplementary material for: Transcriptome Reveals the Effects of Early Weaning on Lipid Metabolism and Liver Health of Yangtze Sturgeon (Acipenser dabryanus)
Source: Int J Mol Sci. 2022 Sep 17;23(18):10866. doi: 10.3390/ijms231810866 (PMC9504784; doi:10.3390/ijms231810866)
Supplement: Supplementary file 1 [file ijms-23-10866-s001.zip › Figure S1-S5.pdf]

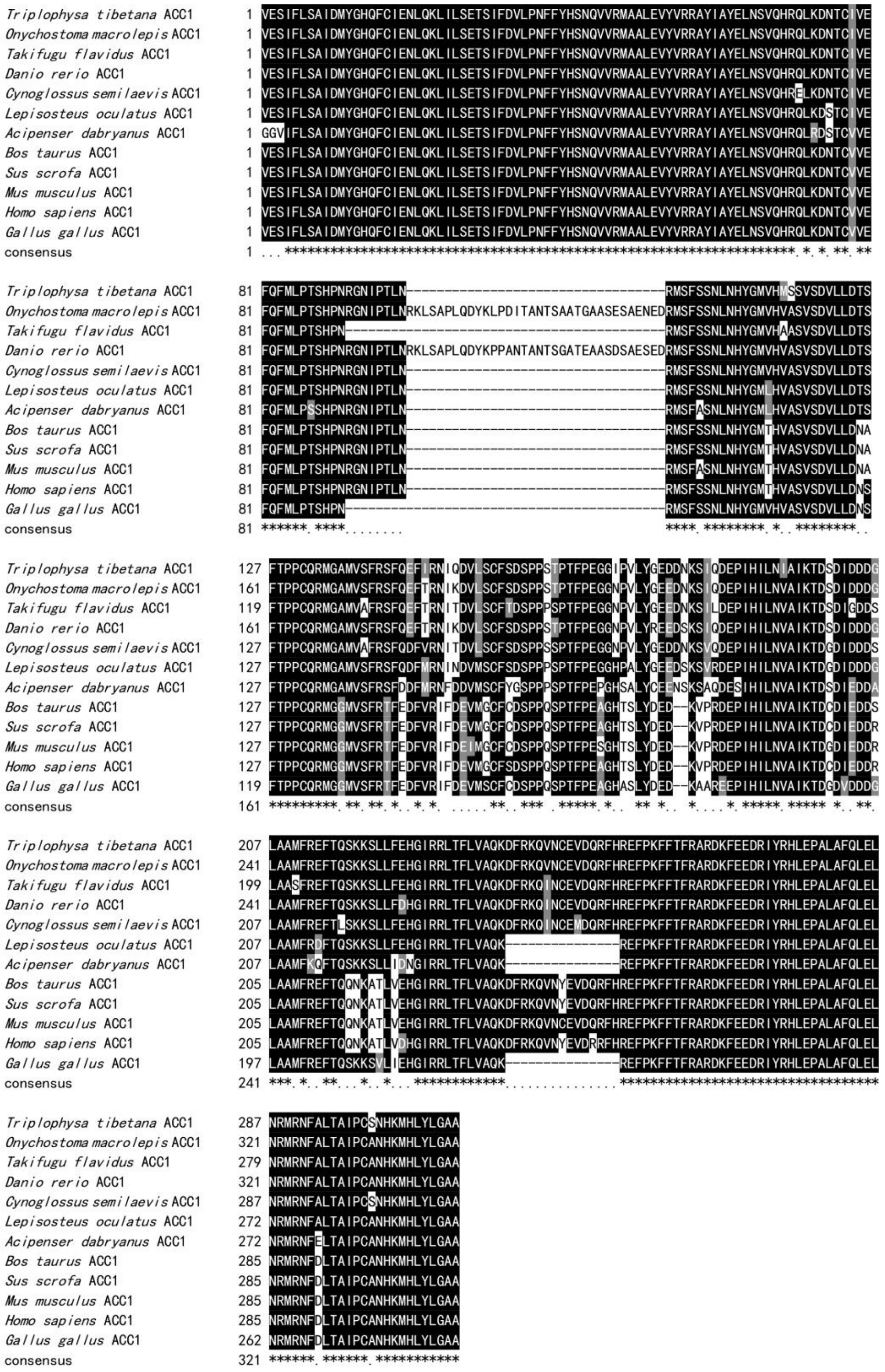

Figure S1. Alignment of the amino acid sequences of *ACC1*. Different gray intensities indicate the conservation of the ammonia acids between species of the five genes. Identical residues are shaded in black, and residues shared by > 33% of the sequences are shaded in gray. * means that these amino acids are consistent.

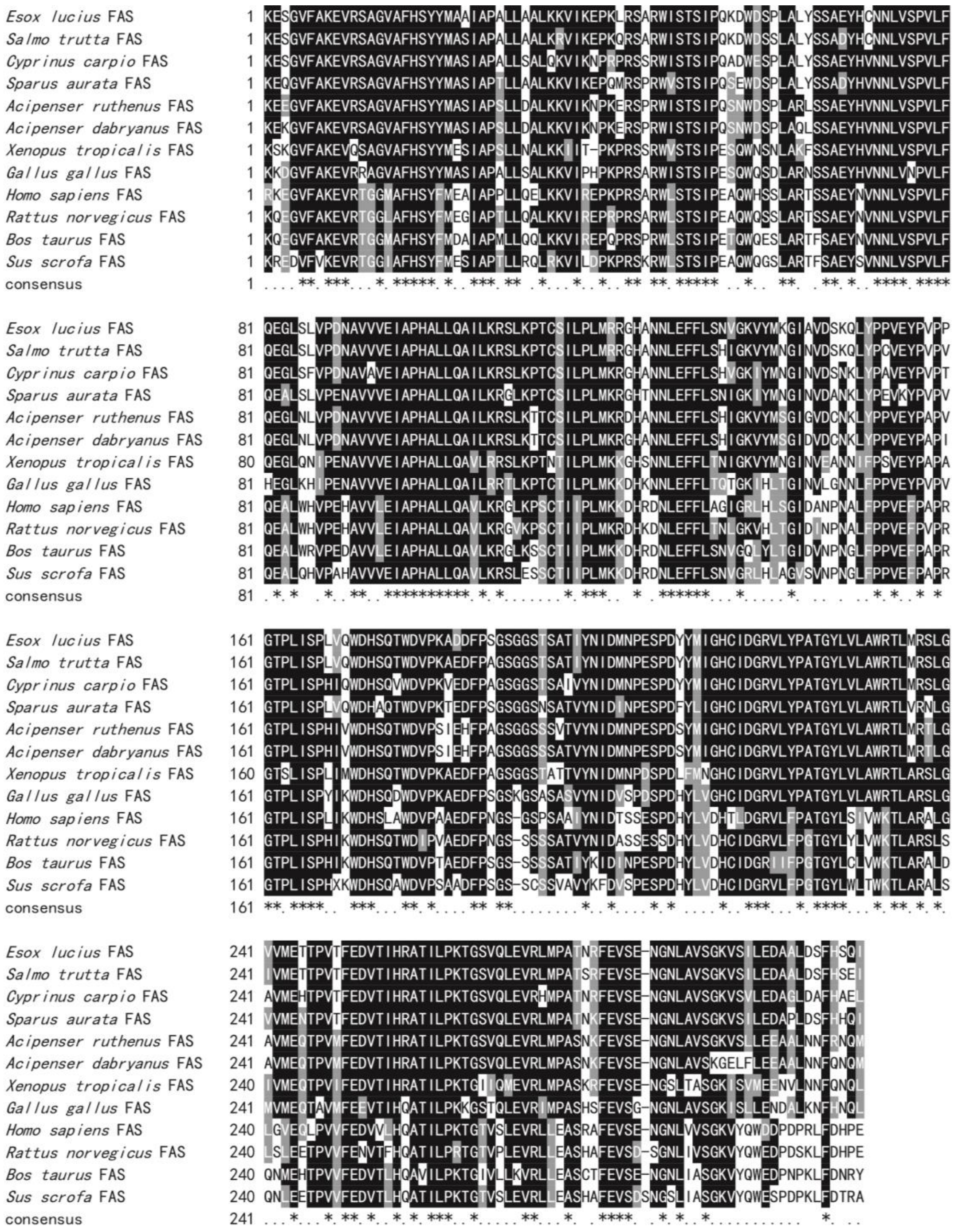

Figure S2. Alignment of the amino acid sequences of *FAS*. Different gray intensities indicate the conservation of the ammonia acids between species of the five genes. Identical residues are shaded in black, and residues shared by > 33% of the sequences are shaded in gray. * means that these amino acids are consistent.

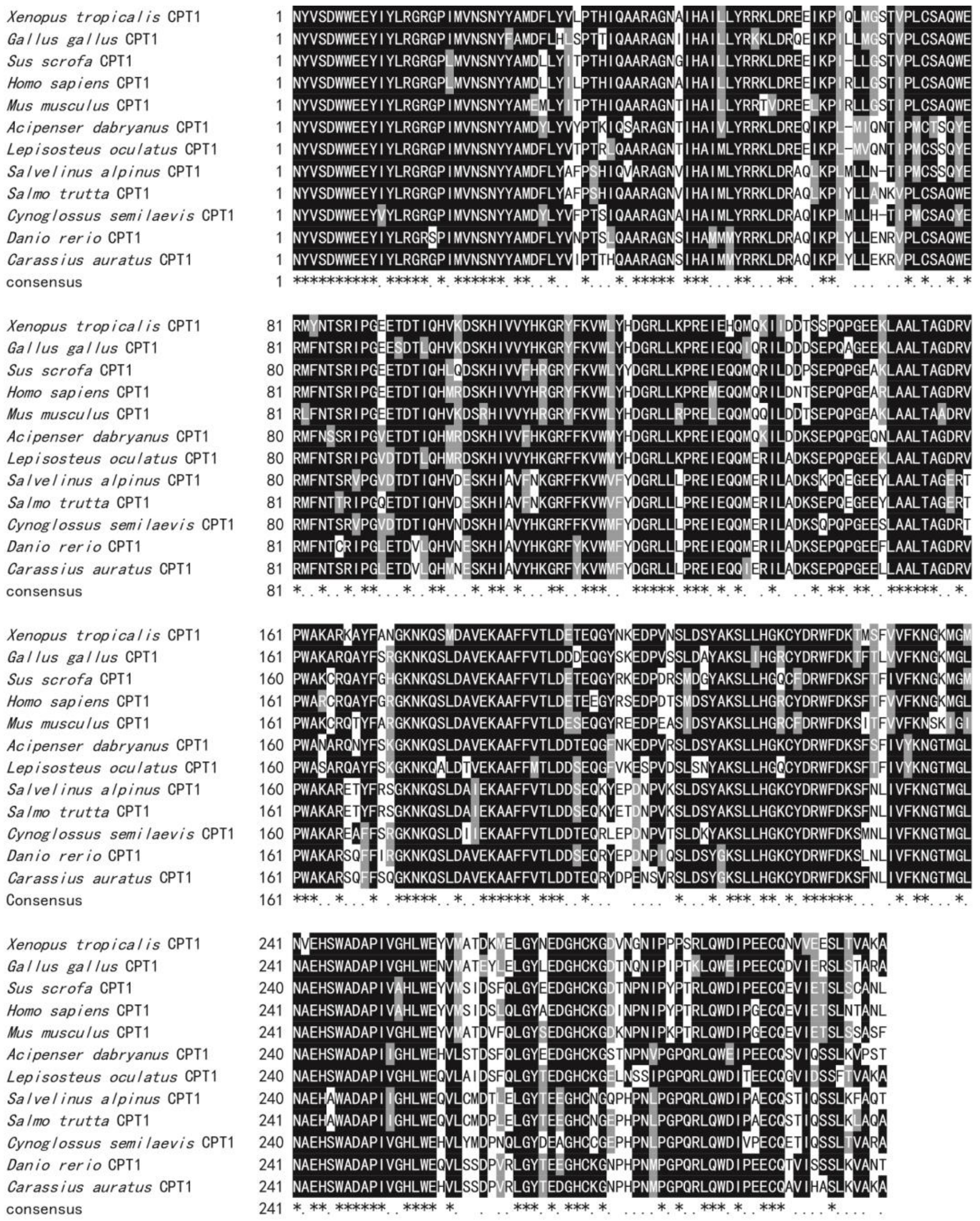

Figure S3. Alignment of the amino acid sequences of *CPT1*. Different gray intensities indicate the conservation of the ammonia acids between species of the five genes. Identical residues are shaded in black, and residues shared by > 33% of the sequences are shaded in gray. * means that these amino acids are consistent.

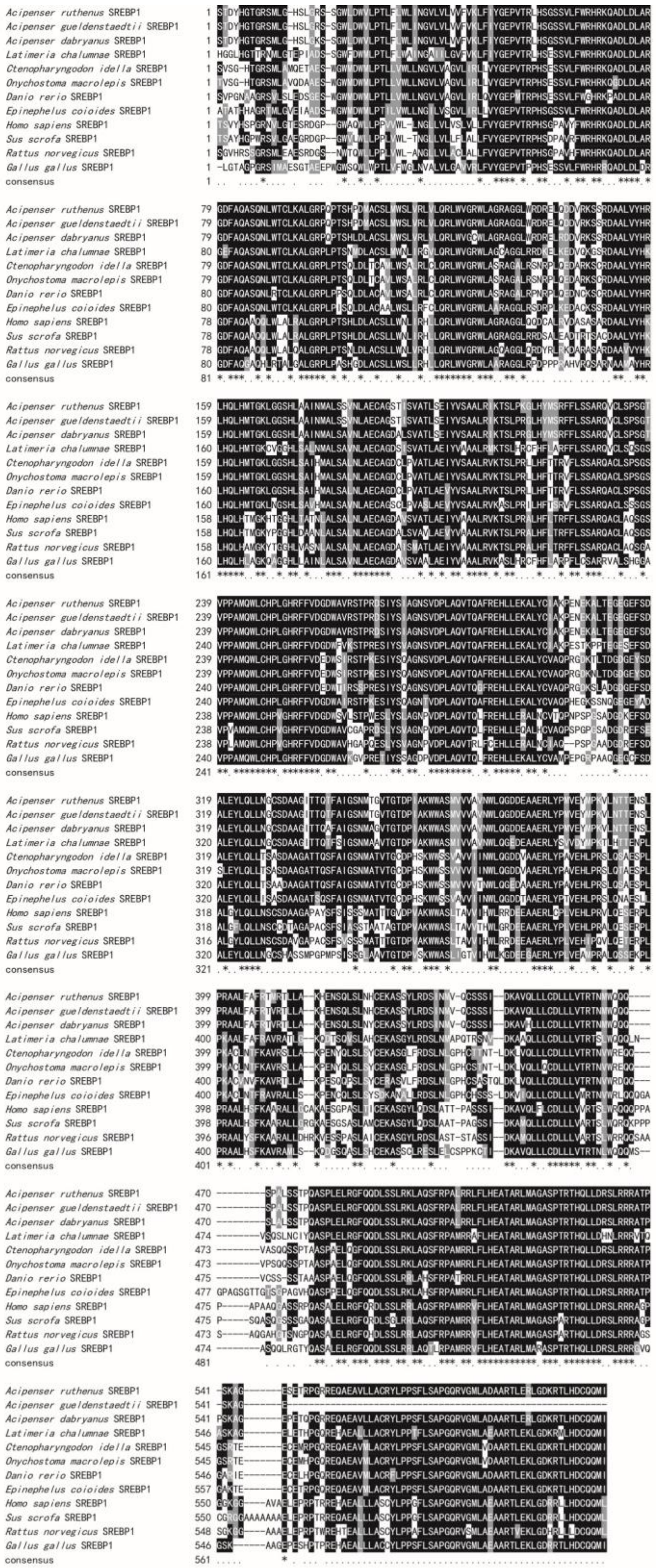

Figure S4. Alignment of the amino acid sequences of *SREBP1*. Different gray intensities indicate the conservation of the ammonia acids between species of the five genes. Identical residues are shaded in black, and residues shared by > 33% of the sequences are shaded in gray. * means that these amino acids are consistent.

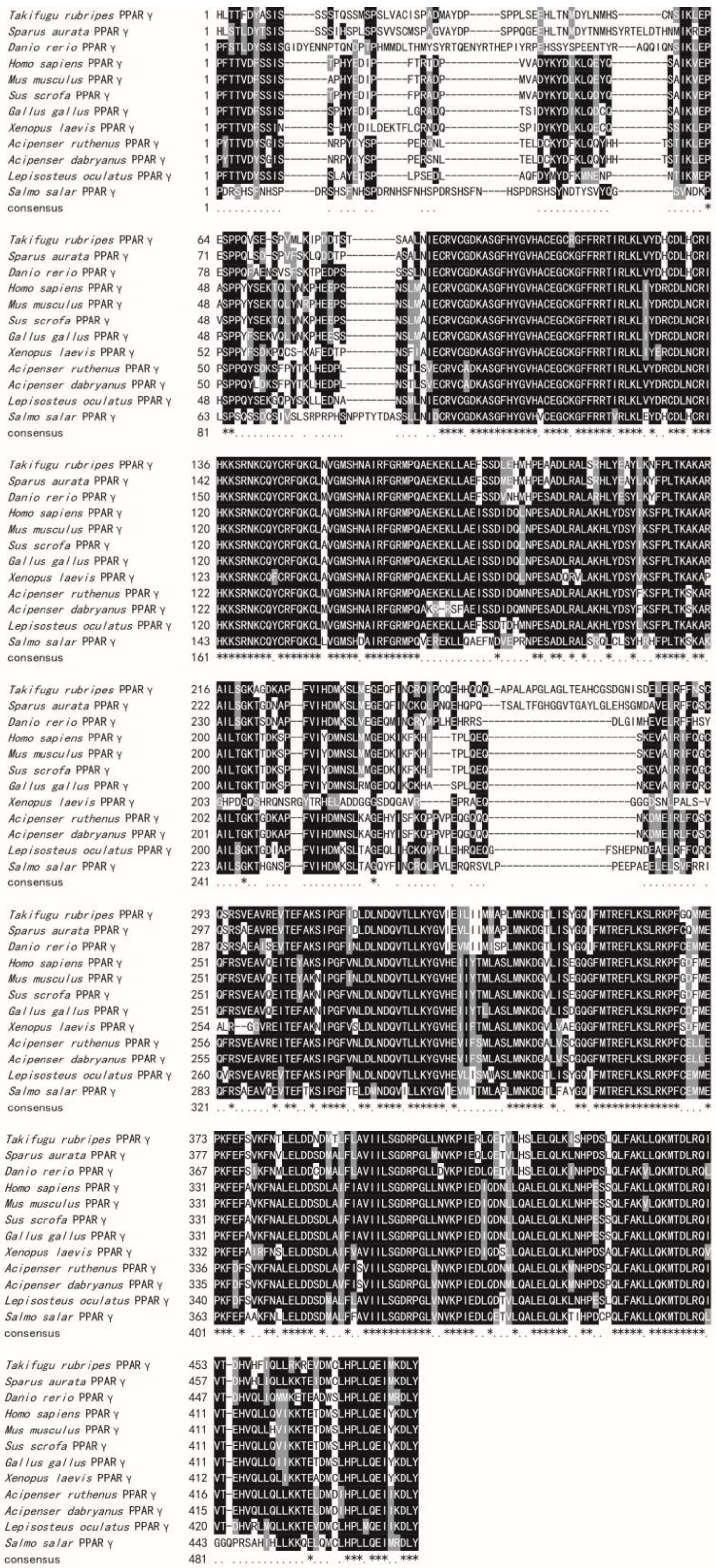

Figure S5. Alignment of the amino acid sequences of *PPARγ*. Different gray intensities indicate the conservation of the ammonia acids between species of the five genes. Identical residues are shaded in black, and residues shared by > 33% of the sequences are shaded in gray. * means that these amino acids are consistent.
