# Supplementary material for: Transcriptome Reveals the Effects of Early Weaning on Lipid Metabolism and Liver Health of Yangtze Sturgeon (Acipenser dabryanus)
Source: Int J Mol Sci. 2022 Sep 17;23(18):10866. doi: 10.3390/ijms231810866 (PMC9504784; doi:10.3390/ijms231810866)
Supplement: Supplementary file 1 [file ijms-23-10866-s001.zip › Table S1.pdf]

Table S1. Sequences of primers used for cloning and qRT-PCR.

| Primer | Sequence | Experiment |
|---|---|---|
| ACC1-F | GCACCTGCCTTCCTATGAG | Clone |
| ACC1-R | CACGAAGAAGCGGTAGTCC | |
| FAS-F | CTGAAGGAAGAGGGCGTC | |
| FAS-R | CATCTGGTTTCGGAAGTTGT | |
| CPT1-F | TTGAAGTGAACCTGGGACC | |
| CPT1-R | GTTGAATGGGAAAGTATGGG | |
| SREBP1-F1 | GCTCTCTGGACAGCACTGATTAT | |
| SREBP1-R1 | TCGGAAAACTCCCCCTCT | |
| SREBP1-F2 | CGCTTCTTTGTGGATGGG | |
| SREBP1-R2 | TTCTCTAAGTGGAGGTGACGG | |
| PPARγ-F1 | CCCTACACGACAGTGGATTA | |
| PPARγ-R1 | CGGGATTCATCTGGTCAA | |
| PPARγ-F2 | GTCACACAACGCCATACGA | |
| PPARγ-R2 | GAAAGGAAAACGGCTACAGA | |
| β-actin-qF | GCCCCACCTGAGCGTAAAT | qPCR |
| β-actin-qR | TCCTGCTTGCTGATCCACAT | |
| AACS-qF | CGATGGGAAAAGAAGGAGACC | |
| AACS-qR | AAGAGAGGGTGACTGAAGGGC | |
| NUCB2-qF | TGGAGACAGACCAGCATTTCAG | |
| NUCB2-qR | GGCTCCGTAACCTGTTCACTTC | |
| junB-qF | ACTCGTTTCTCTCTGCTTATGGC | |
| junB-qR | GCTCGTTCAAGTTCAGGCTCA | |
| c-fos-qF | CCCCATCCTGCTCCACATA | |
| c-fos-qR | TGCTGCTACTGCCTTTCCTG | |
| AKT-qF | CTGATGGCTCTTTCATAGGCTAC | |
| AKT-qR | TGTTTGGCTTTGGTCGTTCT | |
| TNF-α-qF | CAAGATTGTGGTGCCGAGGA | |
| TNF-α-qR | GCAAGTCGCTCGATGTTGTG | |
| EF1-α-qF | ATGTTCACAATGGCAGCGTC | |
| EF1-α-qR | AAGATTGACCGTCGTTCCG | |
| ACC1-qF | CTCACCTTCCTGGTTGCTCA | |
| ACC1-qR | GTGTCTGTAGATCCGATCCTCC | |
| FAS-qF | CCCTAATCTCCCCCCACAT | |
| FAS-qR | GGACACGCCCATCAATACAG | |
| ATGL-qF | ATCACCATCTCACCATTCTCTG | |
| ATGL-qR | ATCCTTGCTGGCACATTTCT | |
| CPT1-qF | TCAACTCCAGTCGCATTCC | |
| CPT1-qR | GGGTTTCAGCAGCCGTC | |
| SREBP1-qF | CACTCGGGCTCCTCTGTTCT | |
| SREBP1-qR | GGGCTTTTAGGCATGTCCAC | |
| PPARγ-qF | GAGCAGGGGCAGCAACAGA | |
| PPARγ-qR | ACCTCGTGGACGCCGTACTT | |
